# T Helper 17 Cells in Primary Sjögren’s Syndrome

**DOI:** 10.3390/jcm6070065

**Published:** 2017-07-05

**Authors:** Kiyoshi Matsui, Hajime Sano

**Affiliations:** Division of Rheumatology, Department of Internal Medicine, Hyogo College of Medicine, Nishinomiya 663-8501, Japan; hsano@hyo-med.ac.jp

**Keywords:** Primary Sjögren’s syndrome, Th17 cells, Treg cells, IL-17, IL-23

## Abstract

Primary Sjögren’s syndrome is an autoimmune disease characterized by diffuse infiltration of lymphocytes into exocrine glands and other tissues. The infiltrating lymphocytes have been identified as subsets of B cells and T cells, including T helper 17 cells, T regulatory cells and follicular helper T cells. The role of these cells in the development of the syndrome is now known, as is their impact on the production of proinflammatory cytokines such as IL-6, IL-17, IL-22 and IL-23. In particular, experimental animal models and patients suggest that a shift in Th17/Treg balance toward the proinflammatory Th17 axis exacerbates primary Sjögren’s syndrome and other autoimmune disorders. Nevertheless, the pathogenesis of the disorder is not yet fully elucidated. This review summarizes the recent advances in therapeutic control of the Treg/Th17 balance, as well as the efficacy of candidate therapeutics against primary Sjögren’s syndrome.

## 1. Introduction

Primary Sjögren’s syndrome is characterized primarily by infiltration of lymphocytes into exocrine tissues such as salivary and lachrymal glands, although infiltration into multiple organs is also observed in at least one-third of patients [1]. T cells constitute > 75% of infiltrating lymphocytes and most of these are CD4 T helper (Th) cells [2], which, we note, are classified as Th1 and Th2 cells, based on the cytokines produced [3]. The syndrome develops when antigen receptors in these T cells are engaged, and thus trigger cytokine secretion and chronic inflammation. Indeed, insights into Sjögren’s syndrome have been inferred from cytokine production in lesional and circulating T cells, especially since the Th1/Th2 paradigm emerged in the 1990s. For example, when initiating events occur in genetically susceptible patients, dendritic cells produce type1 interferons and IL-12 to stimulate IFN-γ production in natural killer and Th1 cells [4]. However, in vitro and in vivo experiments indicate contradictory roles for Th1 and Th2 cells in primary Sjögren’s syndrome although T cells that react against the M3 muscarinic acetylcholine receptor (M3R), which is expressed in exocrine glands and is essential for exocrine secretion, were detected in the peripheral blood of 40% of a cohort of patients. Autoantibodies against this receptor were also circulating in 9%–100% of the same patients [5], highlighting the significance of this receptor in pathogenesis.

In the last decade, a number of Th cells lineages have been identified, including Th0, Th17, regulatory T (Treg) and follicular helper T (Tfh) cells [6]. Of these, Th17 produces IL-17, an archetypal example of the new generation of proinflammatory cytokines [7]. These linages may also interact with B cells, which produce autoantibodies upon activation by type 1 interferons via B cells’ activating factor (also known as TNFLSF13B or BLyS) [6]. Activated B cells and dendric cells also produced IL-6, which, in conjunction with type I interferons, promotes the differentiation of Th17 cells and Tfh cells. In turn, Tfh cells mediate the formation of ectopic formation of germinal centers in salivary glands and intensify autoantibody production by B cells [8]. Th17 cells also coordinate with B cells, to sustain inflammation [9].

**Figure 1:**
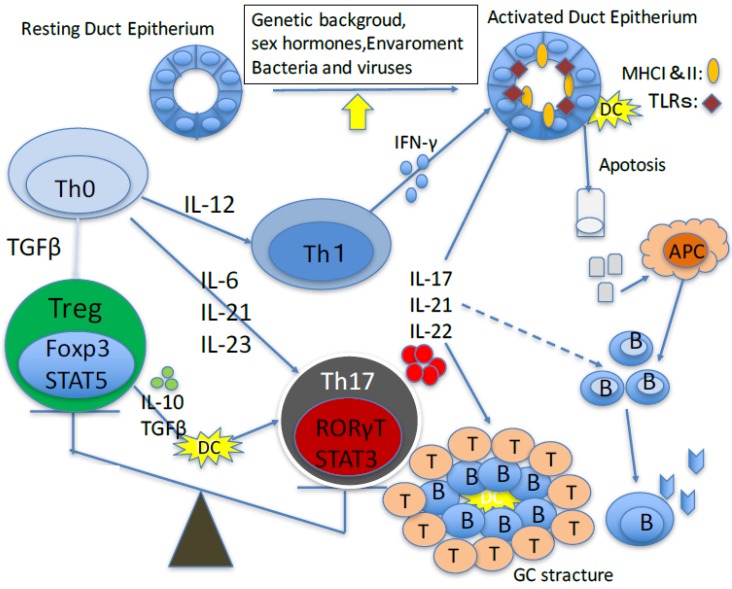
Cells and molecules that derive the pathogenesis of primary Sjögren’s syndrome. APC: antigen presenting cells; DC: dendritic cells; GC: germinal center; IFN: interferon; IL: Interleukin; PC: plasma cells; TGF: Transforming growth factor; Th: T helper cell; MHC: major histocompatibility complex; TLR: Toll-like receptor.

This review summarizes the recent advances in therapeutic control of the Th17/Treg balance, as well as the efficacy of candidate therapeutics against Sjögren’s syndrome (Figure 1).

## 2. Role of Th17 Cells in Animal Models of Primary Sjögren’s Sydrome

IL-2Rα(CD25) knockout mice develop autoimmunity and lymphoproliferative disorders, including autoimmune lacrimal-keratoconjunctivitis that resembles Sjögren’s syndrome. These mice produce significantly higher levels of IL-6, TGF-β1, IL-23R, IL-17A, IL-17F, IL-21, IL-10 and IFN-γmRNA in the cornea and conjunctiva. A mix of Th-1 and Th-17 cytokines is also present with peak production of IL-17 coinciding with peak severity of corneal epithelial disease [10]. In the non-obese diabetic model (NOD) of Sjögren’s syndrome, IL-4 seems to be important in pathogenesis. Indeed, comparable focal infiltrates in the salivary glands are observed in NOD mice deficient in IL-4 and in conventional NOD mice [11]. On the other hand, IL-17 deficiency in B6.NOD-Ace1Ace2 mice, a model of spontaneous Sjögren’s syndrome reduces disease pathology [12], improves glandular function and restores the saliva flow rate [13].

In general, IL-17 is a key mediator of physiological and pathological processes, including in cancer and autoimmunity. For example, IL-17 knockout mice are less susceptible to autoimmune disorders such as collagen-induced arthritis, autoimmune encephalomyelitis and type 1 diabetes [14]. In addition, adoptive transfer of Th17 cells into IL-17 knock out mice induces focal sialadenitis as in wild type mice, underscoring the pathogenic role of IL-17 in primary Sjögren’s syndrome [15]. We note, however, that the knockout mice do not develop primary Sjögren’s syndrome after immunization with a salivary gland peptide. 

On the other hand, Rag1 knockout mice inoculated with splenocytes from M3R knockout mice that had been immunized with synthetic M3R peptides, develop severe Sjögren-like sialadenitis [16]. We note that cell transfers from M3R knockout x IFN-γ knockout mice and M3R knockout x IL-17 knockout mice suggest that IFN-γ and IL-17 are key agents of sialadenitis [5]. 

## 3. Role of Th17 Cells in Primary Sjögren’s Syndrome

Th17 and Treg cells are linked through a web of cytokines, notably TGFβ. Of note, Treg cells are thought to suppress Th17-mediated cellular immunity. Accordingly, Foxp3 staining for Treg cells in minor salivary glands is directly correlated with either focus score or Tarpley’s score [17,18]. In vitro functional assays also indicate that suppressive activity of peripheral blood CD25^high^ cells seems to be preserved in primary Sjögren’s syndrome [19]. 

The notion that only a direct set of Th cells that secrete IL-17 and other cytokines is involved in multiple autoimmune and inflammatory diseases eventually led to a re-examination of infiltrating Treg cells in Sjögren’s syndrome. These new surveys confirmed that IL-17 is consistently expressed in the periductal infiltrates of all minor salivary glands from patients with primary Sjögren’s syndrome, with level of expression correlated with severity of glandular inflammation [20,21]. Expression of IL-23 and IL-22 was also observed [22], of which the latter is derived mostly from natural killer T and Th17 cells. IL-17 and the related cytokines TGFβ, IL-6, IL-23 and IL-12 but not IL-10 were also detected in patient plasma and saliva [21]. Accordingly, peripheral blood cells from patients with Sjögren’s syndrome may have the capacity to abundantly secrete IL-17 to promote Th17 polarization as well as IL-12 to promote Th1 differentiation. IL-18 may also stimulate local IL-17 production in the inflamed salivary gland and thereby boost IgG1 [23].

The activation of Th17 cells in Sjögren’s syndrome infiltrate has been hypothesized to promote B cell activation and formation of germinal centers within glands and to be suppressed by B cell-activation factor [6]. The same Th17 polarization is also observed in inflammatory lesions in the intestinal mucosa in a number of autoimmune diseases [24]. In humans, Th17 polarization is also mediated by retinoic acid-related orphan receptor-γT transcription factor and is induced by local exposure to TGFβ, IL-23, IL-21 and IL-6 [16]. Of these, IL-21 is considered to be a key activator of B and Th17 cells [25] and an attenuator of peripheral Treg cells [26]. Accordingly, IL-21 expression in the serum and labial salivary glands is associated with hypergammaglobulinemia and autoimmunity in patients with Sjögren’s syndrome [27]. We note, however, that evaluation of Treg and Th17 activity is limited by a host of intrinsic factors that deserve some consideration. For example, activated T cells with Th1 and Th2 phenotypes also arise even though Th17 and Th21 cells are predominant and are likely to stimulate B lymphocytes. Further, we note that primary Sjögren’s syndrome is a heterogeneous disease associated with different genetic backgrounds and serological status, and presents a wide spectrum of clinical symptoms. 

On the other hand, Tfh cells organize the germinal center in secondary lymphoid organs [28]. These cells are a specialized subset of CD4^+^ T cells that abundantly express affinity-matured antibodies, T cell co-stimulator and programmed cell death protein1, a T cell co-inhibitor [29]. Of note, expression of C-X-C-motif chemokine receptor 5, a receptor for C-X-C-motif chemokine ligand13, is useful as a marker to identify the subsets of Tfh cells involved in specific processes [28]. 

## 4. Therapeutic Approaches 

The role of B cells is already well-defined in the development of ectopic lymphoid tissue autoimmunity, hypergammaglobulinemia and in some cases, lymphoma [30]. Accordingly, rituximab, a chimeric monoclonal antibody against the B cell antigen CD20, seems to be the most effective biologic against Sjögren’s syndrome dryness [31,32,33]. Strikingly, a recent retrospective survey of the Autoimmune and Rituximab registry indicated that the antibody is typically used in primary Sjögren’s syndrome with systemic organ involvement but rarely in patients with glandular involvement [34]. Thus, the data indicate that, at present, there is only limited use of biologics against primary Sjögren’s syndrome, even though the efficacy of TNF blockers remains in question.

Notably, research over the last 30 years has grown beyond phenomenology, and has an important role for the T cells are activated in response to an environmental trigger in the etiology and pathogenesis of primary Sjögren’s syndrome.Consequently, drugs that prevented T cell activation may be of therapeutic value. For example, administration of abatacept (CTLA4-Ig)—which is a biologic that specifically binds to CD80 and CD86 in antigen-presenting cells, blocks interactions with CD28 on T cells and inhibits a co-stimulation step required to complete T cell activation—is a new therapeutic strategy against autoimmune diseases, such as rheumatoid arthritis [34,35,36,37,38]. Indeed, abatacept was found to mitigate Sjögren-related secretory dysfunction in more patients in recent clinical trials evaluating abatacept in primary Sjögren’s syndrome than in previous trials (ROSE trials) for both rheumatoid arthritis and Sjögren’s syndrome secondary to rheumatoid arthritis [39]. 

In past years, accumulating evidence has highlighted a bias in the Th17/Treg equilibrium toward the pro-inflammatory Th17 program. In turn, the pro-inflammatory Th17 program is assumed to contribute to the pathogenesis of chronic inflammatory including psoriasis, psoriatic arthritis, AS, systemic lupus erythematosus, multiple sclerosis and inflammatory bowel disease [24,40,41].

Thus, various drugs that modulate Treg and Th17 activity have been shown to be therapeutic and have been approved as treatment or are undergoing clinical trials (Table 1). Generally, protocols based on these drugs are multipoint and directly Th17-related cytokines, cytokine receptors and intracellular signaling pathways, inhibit Th17-specific transcription or boost Treg-specific transcription. Indeed, three monoclonal antibodies against Il-17 have been developed in recent years with excellent therapeutic effects, including secukinumab (AIN457), ixekizumab (LY2439821) and brodalumab (AMG827). Secukinumab is now approved against psoriasis, psoriatic arthritis and AS, while ixekizumab is approved against psoriasis [42,43,44,45,46,47,48]. Clinical trials evaluating secukinumab as treatment for dry eye is currently ongoing (trial NCT01250171). 

IL-23 is a heterodimer of two subunits, p19 and p40, of which the latter is also incorporated into the Th1-inducing cytokine IL-12 [49]. In patients with psoriasis, p40 and p19 mRNAs are more abundant in affected skin whereas mRNA levels of p35—the other IL-12 subunit—is less abundantly expressed [50]. Therefore, blocking IL-23 and its cognate receptor IL-23R is another promising therapeutic strategy to inhibit Th17-based autoimmunity. Accordingly, several therapeutic antibodies have been developed including the p40 antibodies ustekinumab and briakinumab. Owing to its favorable efficacy, ustekinumab is already approved against psoriasis [51,52] and psoriatic arthritis [53,54,55,56]. Long-term efficacy has also been reported for ustekinumab, not only against paradoxical forms of psoriasis induced by drug against TNF-α, but also against articular involvement in a patient affected by rheumatoid arthritis and in another affected by Sjögren’s syndrome [57]. Several antibodies against IL-23p19, e.g., tildarakizumab, guselkumab, BI-655066, AMG139, and LY3074828 are in clinical development. These antibodies enable specific targeting of IL-23 without the cross-reactivity against IL-12. While these are currently in phase III clinical trials for psoriasis and in a phase II trials for psoriatic arthritis, they have not been tested against primary Sjögren’s syndrome.

Molecules that regulate the balance between Treg and Th17 are also potential therapeutic targets. For instance, tosilizumab—a humanized antibody against IL-6R—is now undergoing clinical trials against primary Sjögren’s syndrome (NCT01782235). Similar molecules that interfere with sirukumab, olokizumab and clazakizumab are in clinical trials as alternative therapies against rheumatoid arthritis. Other intriguing therapeutics attracting major research interest are potential inhibitors of the retinoic acid-related orphan receptor γT which is considered to be a master transcriptional regulator of the Th17 lineage. Notably, digoxin, which is already well established in cardiology, was found to suppress the differentiation of murine Th17 cells without affecting other T cell linages [59] and to inhibit IL-17 production from T cells in experimental autoimmune encephalomyelitis mice [58].

## 5. Conclusions

Studies of pathogenesis of primary Sjögren’s syndrome highlight a shift in the Th17/Treg balance toward proinflammatory Th17—an event that remains to be fully elucidated. Nevertheless, substantial progress in understanding the development, function and reciprocal regulation of Th17 and Treg cells suggests strong therapeutic potential in targeting these cells. However, the heterogeneity of primary Sjögren’s syndrome in terms of genetic basis, clinical manifestations and serological status may require different therapeutic approaches. Unfortunately, most clinical studies do not categorize patients based on disease features, including disease duration or therapy, and the data may thus be biased. Furthermore, most data have been collected from peripheral blood rather than target organs, but it was recently shown that the pool of Th17 cells is not homogeneous and consists of a non-pathogenic and pathogenic subset [60,61,62]. Hence, targeting only pathogenic Th17-cells could be the next advance in therapies that target Th17/Treg. Indeed, various novel candidate molecules that correct a Th17/Treg imbalance have recently shown promise in animal models [58,63]. Nevertheless, multiple challenges remain in the development and deployment of T17/Treg-targeted interventions against primary Sjögren’s syndrome.

## Figures and Tables

**Table 1:** Therapeutic tools against primary Sjögren’s syndrome. Listed are therapeutics that target Th17 cytokines, cytokine receptors and transcription factors and thus may correct the Th17/Treg imbalance associated with the syndrome.

Target		Drug	Reference or Clinical Trail
cell	B cells	rituximab	[31,32,33]
	T cells	abatacept	[39]
Cytokine & Cytokine and Receptor	IL-6R	tocilizumab	NCT01782235
	IL-17	secukinumab (ixekizumab) (brodalumab)	NCT01250171
	IL-12/23p40	ustekinumab	[57]
	IL-23p19	(tildarakizumab) (guselkumab)	-
Transcription factor RORγT		digoxin	mouse model [58]
